# Melatonin Function and Crosstalk with Other Phytohormones under Normal and Stressful Conditions

**DOI:** 10.3390/genes13101699

**Published:** 2022-09-22

**Authors:** Murtaza Khan, Sajid Ali, Hakim Manghwar, Saddam Saqib, Fazal Ullah, Asma Ayaz, Wajid Zaman

**Affiliations:** 1 Department of Horticulture and Life Science, Yeungnam University, Gyeongsan 38541, Korea; 2 Lushan Botanical Garden, Chinese Academy of Sciences, Jiujiang 332000, China; 3 State Key Laboratory of Systematic and Evolutionary Botany, Institute of Botany, Chinese Academy of Sciences, Beijing 100093, China; 4 University of Chinese Academy of Sciences, Beijing 100049, China; 5 State Key Laboratory of Grassland Agro-Ecosystems, School of Life Sciences, Lanzhou University, Lanzhou 730000, China; 6 State Key Laboratory of Biocatalysis and Enzyme Engineering, School of Life Sciences, Hubei University, Wuhan 430062, China; 7 Department of Life Sciences, Yeungnam University, Gyeongsan 38541, Korea

**Keywords:** melatonin, phytohormone, biotic stress, abiotic stress, plant growth, crop improvement, stress mitigation

## Abstract

Melatonin was discovered in plants in the late nineties, but its role, signaling, and crosstalk with other phytohormones remain unknown. Research on melatonin in plants has risen dramatically in recent years and the role of this putative plant hormone under biotic and abiotic stress conditions has been reported. In the present review, we discuss the main functions of melatonin in the growth and development of plants, its role under abiotic stresses, such as water stress (waterlogging and drought), extreme temperature (low and high), salinity, heavy metal, and light-induced stress. Similarly, we also discuss the role of melatonin under biotic stresses (antiviral, antibacterial, and antifungal effects). Moreover, the present review meticulously discusses the crosstalk of melatonin with other phytohormones such as auxins, gibberellic acids, cytokinins, ethylene, and salicylic acid under normal and stressful conditions and reports melatonin receptors and signaling in plants. All these aspects of melatonin suggest that phytomelatonin is a key player in crop improvement and biotic and abiotic stress regulation.

## 1. Introduction

Plants are exposed to a variety of environmental stresses (biotic and abiotic) during the course of development. Stressful conditions alter the basic metabolism of the affected plants. Plants must cope with environmental constraints to effectively complete their life cycle [1,2]. Plant produce and regulate various biomolecules to adapt to adverse environmental conditions [3,4,5]. One of the most studied molecules is melatonin, which acts as an effective protective agent against stressful conditions. Melatonin (N-acetyl-5-methoxytryptamine) is a ubiquitous molecule that is present in plants, animals, and microorganisms. It has been revealed as an indolic chemical compound with structural similarities with other vital compounds such as tryptophan, serotonin, and indole-3-acetic acid (IAA) [6,7]. Melatonin was discovered in animals in 1958, in microbes in 1991, and then in plants in 1995 [8]. After its discovery, melatonin research was mostly conducted on animals for four decades. The pivotal roles of melatonin in the regulation of the antioxidant system, circadian rhythms, cardiac disease, Alzheimer’s disease, and physical health and emotional status were reported and provided the basis for future research directions [9]. Similarly, the social, economic, and other physical health-related benefits include the regulation of jet lag and immunity, sleep promotion, and its antitumor and antiaging characteristics [10,11] in animals. In plants, melatonin is a putative hormone involved in the regulation of plant growth and productivity, even under biotic and abiotic stress conditions [12].

The discovery of melatonin in plants paved the way for its understanding and revealed that melatonin is a common and multipurpose metabolite in the plant world. It is found in almost all parts of the plants, including the leaves, stems, roots, flowers, fruits, and seeds of numerous plants [8]. It is involved in the regulation of plant growth, leaf development, root organogenesis, fruit maturation, and senescence [13]. Furthermore, it significantly contributes to the responses of plants to environmental stresses including heat, salinity, drought, oxidative stress, and ultraviolet-B (UV-B) radiation [14].

**Figure 1:**
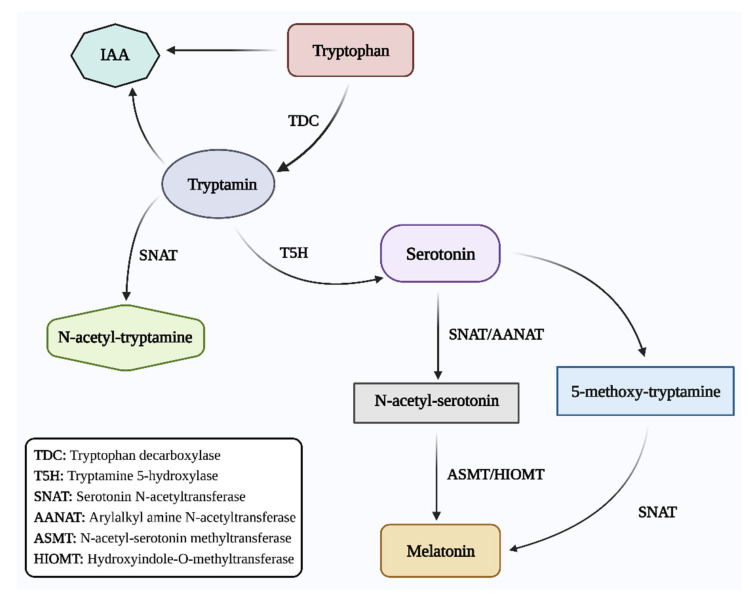
Schematic pathway of melatonin expression. This figure was created with BioRender.com (accessed on 15 September 2022).

In plants, melatonin production can be induced by a variety of conditions, including light, temperature extremes, and UV-B radiation [14]. Tryptophan serves as the precursor for the production of melatonin in a variety of plants. Tryptophan decarboxylase (TDC) catalyzes its conversion to tryptamine, which is then turned to serotonin by the enzyme tryptamine 5-hydroxylase (T5H) [15]. Serotonin N-acetyltransferase (SNAT)/arylalkyl amine N-acetyltransferase (AANAT) converts serotonin into N-acetyl serotonin. N-acetyl serotonin is converted into melatonin by the action of N-acetyl-serotonin methyltransferase (ASMT)/hydroxyindole-O-methyltransferase (HIOMT). In addition, SNAT can catalyze the conversion of tryptamine into N-acetyl-tryptamine (Figure 1). However, T5H cannot convert it into N-acetyl-serotonin. Whether there is a mechanism for turning N-acetyl-tryptamine into N-acetyl-serotonin is unclear. The second pathway involves the enzyme HIOMT, which changes serotonin into 5-methoxy-tryptamine, and the enzyme SNAT, which transfers 5-methoxy-tryptamine into melatonin [16]. Furthermore, in the reverse melatonin pathway, N-acetyl-serotonin deacetylase converts N-acetyl-serotonin into serotonin [17]. Additionally, tryptophan is a precursor for both melatonin and IAA, which indicates that melatonin has several functions in plants.

In the present review, the role of melatonin in plants under biotic and abiotic stress conditions is discussed extensively. The novelty of the present work is based on reporting the relationships between melatonin and other phytohormones with special emphasis on the signaling and receptors in plants.

## 2. Role of Melatonin in Plant Growth and Development

**Figure 2:**
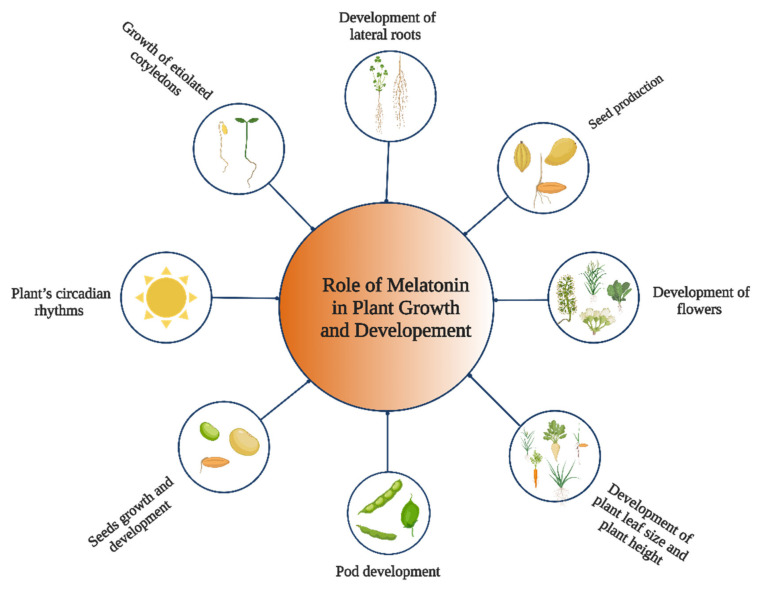
Role of melatonin in plant growth and development. This figure was created with BioRender.com (accessed on 7 September 2022).

Several phytohormones, mainly auxin, play a crucial role in the growth and development of plants. Melatonin and indole acetic acid share the same precursor, tryptophan, making them both types of indoleamines. As such, melatonin should be involved in the control of plant growth and development (Figure 2). Previous results indicated that melatonin regulates the plant’s circadian rhythms in the *Chenopodium rubrum* [18]. Furthermore, in *Chenopodium rubrum,* melatonin’s application also affected the development of flowers in the early stage of the photoperiod [19]. After being treated with melatonin, the soybean plant’s leaf size, plant height, pod size, and production of seeds all dramatically increased, indicating that the application of melatonin may enhance the soybean plant’s growth and seed production [20]. Melatonin’s shielding effect in the senescence process of plants was shown by the fact that it reduced the breakdown of chlorophyll in the leaves of barley plants [21]. Melatonin could encourage the growth of etiolated cotyledons in *Lupinus albus*, which is similar to how IAA works [22].

Additionally, melatonin’s effects on plants vary depending on its concentration. Low melatonin concentrations (10–20 µM) exhibited no discernible impact on root length in Arabidopsis seedlings. On the other hand, fresh weight at high melatonin content (200–400 µM) was greatly suppressed, and the ideal melatonin level for promoting plant growth and development was 40 µM [23].

## 3. Role of Melatonin under Abiotic Stress

**Figure 3:**
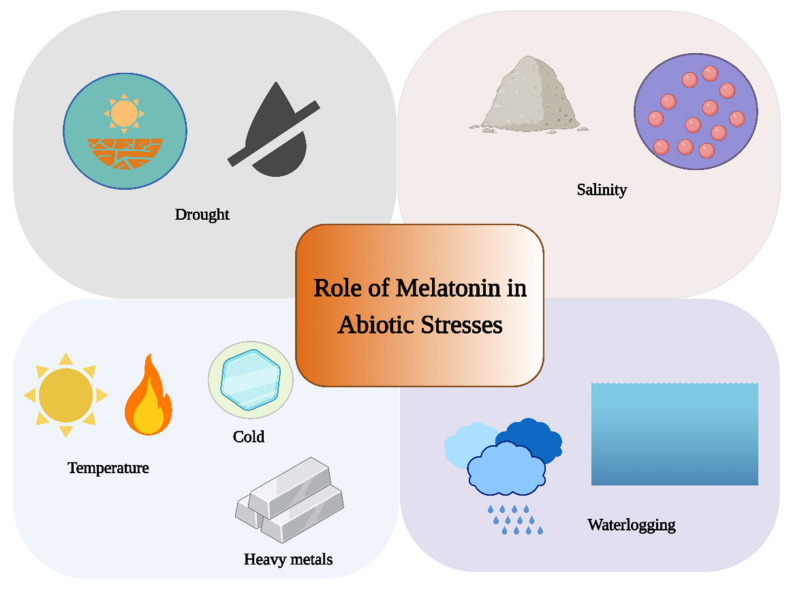
Role of melatonin under abiotic stress. This figure was created with BioRender.com (accessed on 7 September 2022).

Throughout their existence, plants are subject to a variety of environmental pressures. Plants, which are sessile organisms, have developed a variety of coping mechanisms to deal with challenging situations, maintaining their survival and ability to reproduce [24,25]. Melatonin is a universal abiotic stress regulator in plants [6]. Exogenously applied melatonin increases plant tolerance against abiotic stresses, including drought, waterlogging, extreme temperatures, salinity, and heavy metals toxicity, by modifying the production of endogenous melatonin and antioxidant systems [26] (Figure 3).

### 3.1. Role of Melatonin under Water Stress

Drought stress dramatically reduces plant growth and development [27]. The morphological, physiological, biochemical, and molecular properties of plants are altered by drought stress, which poses a major threat to agricultural productivity and quality [28]. Under abiotic stress, endogenous melatonin levels are increased [29]. The Arabidopsis plant’s ability to withstand drought was significantly improved by the overexpression of the melatonin production gene, MzASMT1 [30]. Thus, exogenous melatonin could be used to alleviate abiotic stresses. For example, melatonin application improved drought tolerance in drought-sensitive and drought-resistant species of apple plants [31]. Similarly, melatonin supplementation reduced the adverse effects of drought stress on the photosynthetic and antioxidant systems of grapes [32].

Waterlogging adversely affects plant growth and development. This process substantially restricts gas diffusion, causing hypoxic stress brought on by anaerobic respiration in the roots and encouraging the buildup of reactive oxygen species (ROS) [33]. Melatonin takes a role in the control of plant reactions to waterlogging. In plants, in response to waterlogging stress conditions, the transcript accumulation of the genes involved in melatonin is dramatically increased [34]. Under waterlogging conditions, exogenously applied melatonin significantly increased seedling viability in apples [35]. Furthermore, melatonin was found to be able to improve cucumber and *Prunus persica* resistance to waterlogging by stimulating root development, increasing antioxidant enzyme activity, and improving photosynthetic efficiency [35].

### 3.2. Role of Melatonin under Extreme Temperature

Cold stress adversely affects plant growth and survival. It may cause an excessive ROS buildup and redox imbalance. Melatonin tends to accumulate in extreme cold conditions to shield plants from deadly injuries. For instance, melatonin plays a protective role in plants’ ability to withstand low temperatures, as shown by SNAT transgenic rice, which is less sensitive to cold than wild-type plants [36]. Exogenously applied melatonin may improve the cold and drought tolerance of tobacco, tomato, and cucumber [37]. The application of melatonin significantly enhanced the germination rate of cucumber seeds from 4% to 83% at 10 °C [38]. Wheat seedlings supplemented with melatonin showed increased levels of osmoprotectants and antioxidant enzyme activity, indicating that melatonin may increase the plant’s ability to withstand low temperatures by scavenging ROS and regulating redox equilibrium [39]. Exogenously applied melatonin can sustain the quality of fruits, vegetables, and cut flowers by conferring chilling tolerance. For instance, pre-treating loquat fruit with melatonin before storage causes a buildup of phenolic chemicals and a decrease in lignin, relieving taste and nutrient loss brought on by chilling damage when exposed to cold storage [40].

Heat stress also adversely affects the growth and survival of plants and is becoming a worldwide concern because of global warming. Heat stress adversely affects the physical, biochemical, and molecular properties of the plants [41]. Additionally, by raising endogenous melatonin, ASMT and SNAT overexpression dramatically enhances thermotolerance [42]. Melatonin concentration was significantly enhanced when the plants were challenged by heat stress [43]. Thus, melatonin treatment might improve the plant’s resistance to heat stress. Exogenously applied melatonin dramatically improved the germination percentage of *Arabidopsis thaliana* [44]. Melatonin therapy increased heat stress tolerance in tomato seedlings by maintaining redox homeostasis while regulating polyamine and nitric oxide production [45]. Melatonin treatment improved the production of SA and lowered the concentration of ABA in soybean seedlings to decrease fatal heat-induced injuries [46].

### 3.3. Role of Melatonin under Salt Stress

Salt stress has emerged as a serious global issue, restricting agricultural output and causing significant economic losses globally [47]. Melatonin has reportedly been linked to an increase in plants’ resistance to salt stress in recent years. Melatonin treatment increased salt tolerance in several plants, including barley, wheat, cucumbers, soybeans, bermudagrass, and apples [48]. Similarly, in cucumber plants, the adverse effects of salt stress on the root system were significantly reduced via the application of melatonin [49]. Furthermore, melatonin treatment increased salt tolerance and regulated transcript accumulation of the genes related to salt stress [20]. Melatonin application also increased the expression of the genes related to the production and catabolism of abscisic acid (ABA) and gibberellic acid (GA) in cucumber plants during salt-induced stress [50].

### 3.4. Role of Melatonin under Heavy Metal Stress

Pollution from heavy metals (HMs) poses a major threat to all types of living things, notably to plants [51,52]. HMs application, including cadmium, lead, and zinc, significantly increased the production of endogenous melatonin in algae, and exogenous melatonin application improved the algae’s ability to withstand cadmium stress [43,53]. Furthermore, exogenous melatonin application dramatically induced the tolerance of the plants to HMs stress [45]. For example, exogenously applied melatonin substantially reduced the toxicity caused by cadmium in tomatoes [54]. Melatonin and nitrogen oxide interaction enhanced Pb and Cd stress tolerance [48]. Melatonin treatments at concentrations of 1 and 10 µM boosted seed germination and seedling growth when exposed to copper stress, whereas the application of 100 µM melatonin showed opposite effects and increased copper’s harmful effects [55].

Melatonin controls antioxidant levels as well as the uptake and sequestration of heavy metals, which helps to modulate the tolerance to heavy metals. For example, vanadium was excluded or sequestered from the plants via melatonin application [56].

### 3.5. Role of Melatonin under Light-Induced Stress

Plants are harmed by light-induced oxidative bursts. In plants, UV radiation can cause the production of free radicals [57]. After exposure to UV-B light for a brief period, endogenous melatonin concentration was shown to increase in plants, indicating that it plays a role in the UV-B response [58]. Under UV-B exposure, exogenous melatonin increased the number of isoflavone monomers in 4-day-old germinated soybeans [59]. In Arabidopsis, melatonin application induced UV-B tolerance [60].

## 4. Role of Melatonin in Biotic Stress

To combat biotic stressors, plants often have a highly developed immune system. First, physical barriers to plants, including waxes, thick cuticles, and unique trichomes, prevent pathogens or insects from adhering to them [12,61,62]. Plants have two pathways that they can use to recognize pathogens and launch defense mechanisms. The first one is the pattern recognition receptors (PRRs), which recognize pathogen-associated molecular patterns (PAMPs) such as flagellin to induce PAMP-triggered immunity (PTI) [63]. Plant resistance (R) proteins, the second route of the immune system, detect the specific effectors of pests or pathogens (avirulent proteins) and trigger the plant defense response through a mechanism known as effector-triggered immunity (ETI) [63,64]. ETI induces a hypersensitive response (HR), an intentional cell suicide of the infected cells [63]. A number of plant hormones, including ethylene (ET), jasmonic acid (JA), and salicylic acid, are particularly prominent in the signaling pathways induced by PTI and ETI (SA). Plants frequently induce the ET and JA pathways in response to chewing insects and necrotrophic infections, but the SA mechanism enhances resistant protection against hemi-biotrophic and biotrophic pathogens [63,65]. The first is known as systemic acquired resistance (SAR), which becomes active during primary infection with a necrotizing pathogen and is associated with rising concentrations of SA and related pathogenesis proteins [63]. The second type of plant resistance is induced systemic resistance (ISR), which is triggered by particular strains of nonpathogenic root-colonizing bacteria and requires JA and ET for signaling [12,66]. By identifying the conserved herbivore-associated elicitors of the invading insect, phytophagous insects force plants to exhale volatiles to attract their foes and warn their neighbor plants of impending hazards [67,68].

**Figure 4:**
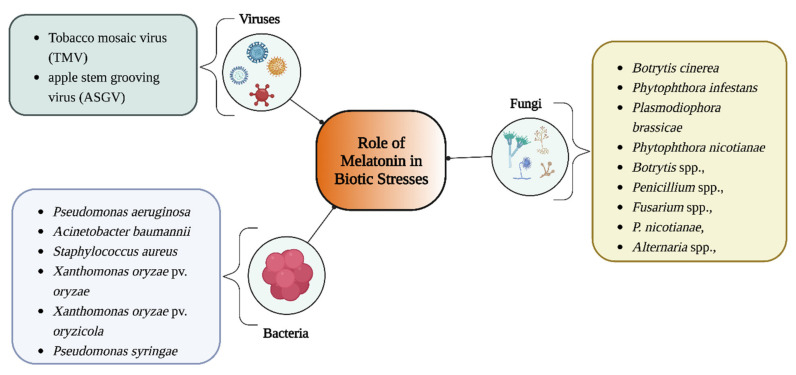
Role of melatonin in biotic stress. This figure was created with BioRender.com (accessed on 7 September 2022).

Melatonin may be a cost-effective alternative method to induce plant protection against biotic stress because it is an eco-friendly chemical (Figure 4). Animal studies have shown that melatonin possesses immunomodulatory, antioxidant, anti-inflammatory, and neuroprotective properties [69], making it a potential therapeutic alternative for the treatment of microbial illnesses. Similarly, several significant discoveries have recently demonstrated the positive role that melatonin plays in plant–pathogen interactions. In this context, extra pertinent information is covered in-depth in the following subsections.

### 4.1. Antiviral Effects of Melatonin

Melatonin’s antiviral activity in animals has been proven in numerous studies. In comparison with infected control mice, melatonin therapy drastically reduced blood and brain viruses [70]. Similarly, mice infected with the influenza virus survived longer when given melatonin along with the antiviral medication ribavirin [71]. Melatonin’s great antioxidation efficacy and capacity to reduce endoplasmic reticulum stress make it a candidate in this situation for regulating the autophagy process during various viral infections [72,73,74]. Few researchers have examined the antiviral properties of melatonin in plants up until this point. Tobacco mosaic virus (TMV) viral RNA and virus concentration were reduced in infected *Nicotiana glutinosa* and *Solanum lycopersicum* seedlings after treatment with exogenous melatonin. The rise in SA concentrations in the NO-dependent pathway was thought to be the cause of melatonin’s beneficial effects [75]. Additionally, the apple stem grooving virus (ASGV) of "Gala" apple shoots that had been infected in vitro was successfully destroyed by melatonin, suggesting that it may be possible to grow plants devoid of viruses [76].

### 4.2. Antibacterial Effects of Melatonin

Both in vitro and in vivo studies have been conducted to examine the defense mechanisms of melatonin against bacterial infections in animals. Melatonin’s ability to kill bacteria that are resistant to many drugs, including carbapenem-resistant *Pseudomonas aeruginosa*, *Acinetobacter baumannii*, and methicillin-resistant *Staphylococcus aureus*, has been demonstrated in vitro [77]. Melatonin application also showed a strong inhibitory action against Mycobacterium TB (H37Rv strain) [12]. Melatonin has demonstrated efficient antibacterial activity against phytobacterial pathogens in plant–bacteria interactions. One study found that melatonin application reduced the occurrence of a bacterial leaf streak (BLS) in rice [78].

Melatonin, along with nitric oxide, increased the transcript accumulation of SA pathway-related genes [79]. Additionally, in *Pseudomonas syringae* pathovar tomato (Pst)-DC3000-infected *Arabidopsis thaliana*, melatonin can trigger MAPK cascades to induce SA production [80]. Transcriptomic data have recently shown that melatonin application triggers ETI- and PTI-associated genes in watermelon and Arabidopsis [81]. Some of the melatonin defense mechanisms against bacteria and fungi are given in Table 1.

### 4.3. Antifungal Effects of Melatonin

Melatonin was shown to have therapeutic advantages in animal models of *Candida sepsis* and conventional antimycotic therapy, where it could reduce interleukin-6 concentrations and shorten the amount of time needed for recovery from *Candida sepsis* in rats [86]. Melatonin promoted tomato fruit resistance to *Botrytis cinerea* by controlling the production of H_2_O_2_ and the jasmonic acid signaling pathway [87]. In watermelon and other cucurbits, a rise in melatonin accumulation in plants increases resistance to foliar diseases, such as powdery mildew and soil-borne oomycetes, through alterations in the transcript accumulation of the genes linked to PTI and ETI [81]. The prevalence of *Plasmodiophora brassicae* infection of *A. thaliana* and the number of pathogen sporangia decreased following melatonin treatment. This decrease was ascribed to the high expression of the JA-responsive *PR3* and *PR4* genes [12].

Melatonin and ethylicin, an oomycete antifungal, work synergistically to prevent the growth of *Phytophthora nicotianae* in vitro and in vivo by disrupting the fungus’ amino acid metabolic homeostasis [88]. Melatonin is exogenously applied to replant soil to promote apple seedling growth, boost potassium levels, and induce photosynthesis, all of which alleviate replant disease [89]. Other fungi, such as *Botrytis* spp., *Penicillium* spp., *Fusarium* spp., *P. nicotianae*, and *Alternaria* spp., also showed similar results [12]. Additionally, several studies have examined the function of endophytic rhizobacteria in enhancing plants’ capacity to synthesize melatonin [90]. A number of different theories have explained melatonin’s preventive function against plant fungal infections. For example, some scientists have suggested that melatonin’s defense mechanism involves its capacity to maintain H_2_O_2_ cellular concentrations and the production and control of antioxidant enzyme activities [85].

Transcriptomic data have recently shown that exogenous melatonin administration activates PTI- and ETI-related genes in watermelon and *A. thaliana* [81]. Additionally, melatonin is essential for controlling the levels of ROS and reactive nitrogen species (RNS) in plants, which are signals for numerous cellular and physiological responses to biotic and abiotic stresses. These responses can be triggered directly by ROS/RNS scavengers or indirectly by genes that control the redox network [91].

## 5. Crosstalk between Melatonin and Other Phytohormones

Researchers reported the interactions of phytomelatonin with other phytohormones [92]. Due to their molecular similarities, auxin (IAA) was the focus of the initial studies on the interaction between melatonin and other plant hormones [93]. However, numerous studies have revealed intriguing connections between melatonin and nearly all known plant hormones, including more recent hormones such as JA, SA, brassinosteroids, polyamines, and strigolactones, as well as classical hormones such as auxin, gibberellin, cytokinins, and ABA [93].

### 5.1. Melatonin and Auxin

The three factors investigated for both melatonin and the auxin IAA are growth capacity, rooting capacity, and gravitropism. The similarities between melatonin and IAA compounds were studied in lupin plants [94]. Additional research revealed that melatonin could induce vegetative growth as well, but via a different mechanism [95]. The amount of melatonin and the kind of tissue both affect growth. For example, root growth is more sensitive than leaf growth. IAA and melatonin both hinder growth at high melatonin levels. Recent investigations have demonstrated that high quantities of melatonin block IAA production, whereas low concentrations of IAA increase it. Recent studies, however, have demonstrated that melatonin regulates the function of signaling elements, including auxin receptors, regulators, and tiny auxin upregulated RNA genes, to activate growth processes [96]. However, the complicated data show a dearth of studies in this area.

Early research with lupin also offered information on melatonin’s capacity to facilitate root growth [97]. Since then, it has been proven that melatonin encourages the growth of lateral and adventitious roots in a variety of different species [95]. Melatonin’s ability to produce lateral and adventitious roots is one of this molecule’s most researched functions, and it is frequently related to auxins such as IAA, 1-naphthaleneacetic acid, and indole-3-butyric acid. The amount and length of adventitious roots and the quantity of new lateral roots are all altered by melatonin. For instance, melatonin was found to boost the development of adventitious roots by twofold and the emergence of lateral roots by up to threefold in *A. thaliana*. However, it did not appear to have any impact on root hair density [98]. Additionally, three overexpressed lines of melatonin in *A. thaliana* showed more lateral roots than the wild type [30]. IAA and melatonin collaborate to create roots in plants. Melatonin is a powerful chemical that regulates root architecture in rice. It dramatically restricted embryonic root growth and encouraged the production and growth of lateral roots, and it increased the expression of several genes in the meristem of the root tip and specific elements of the root [99]. In a similar way, melatonin’s effects on the auxin signaling pathway in rice led to an improvement in the root system [99].

### 5.2. Melatonin and Gibberellic Acids (GAs)

Exogenously applied melatonin increased the transcript accumulation of GA-producing genes in cucumber plants to support germination processes that were impeded by salt [49]. Early research on cucumber and red cabbage showed that melatonin has germination-promoting properties [38,55]. *Brassica napus* L. exposed to salt stress receives melatonin therapy, which stimulates seedling growth by elevating GA levels and upregulating three essential GA production enzymes (GA20ox, GA3ox, and GA2ox). Additionally, GID genes (GA receptors), which encode the soluble GA receptor that joins with GA and DELLA proteins to create a complex that prevents DELLAs from suppressing GA signaling, were upregulated. This results in strong GA signaling, which aids in seedling growth [96]. Similar behavior was seen in rice seedlings raised in fluoride-rich soil [100]. Melatonin treatment boosted the GA concentration and synthesis of cyclin in apple plants [101].

Regarding reproductive development, the application of melatonin slowed down the floral transition in Arabidopsis by increasing the expression of flowering locus C and decreasing the expression of flowering locus T [102]. Melatonin’s direct impact on GA levels is debatable because it has been shown to raise GA levels in response to some stressors [103].

### 5.3. Melatonin and Cytokinins (CKs)

Research on the melatonin–CK interaction has shown that exogenous melatonin therapy raises CK levels: CK was observed to increase the expression of melatonin biosynthesis genes with an increase in endogenous melatonin. Additionally, response transcription factors (ARR, types A and B) and other CK signaling genes showed upregulation. In drought-stressed wild-type and isopentenyl transferase-overexpressing transgenic creeping bentgrass, CKs and melatonin work in concert to improve physiological indices, such as photochemical efficiency, chlorophyll content, and relative water content, increasing drought tolerance [104]. Similar to this, in *B. napus*, the upregulation of numerous CK signaling components, including A-ARR and B-ARR, was linked to an increase in growth following CK-mediated melatonin administration [96]. CK and melatonin have recently been linked to the ripening of sweet cherries [105].

### 5.4. Melatonin and Abscisic Acid (ABA)

In terms of the melatonin–ABA interaction, melatonin administration typically results in a drop in ABA levels, the downregulation of ABA biosynthesis enzymes, and/or a decreased sensitivity to ABA as a result of altered ABA signaling element control. In general, melatonin therapy causes the downregulation of 9-cis-epoxycarotenoid dioxygenase (NCED), a crucial enzyme in ABA biosynthesis, and the upregulation of ABA catabolism genes (CYP707 monooxygenases), resulting in a rapid fall in ABA level. Cucumbers, apples, Chinese cabbage, and hickory are suitable examples of this [106]. The stress conditions that are given to the plants and the melatonin concentration employed, however, have a significant impact on this response. However, in some plants, including barley and *Elymus nutans*, the reverse reaction takes place (higher ABA levels) [106]. A recent study on melatonin-treated mango fruits showed a delay in ripening and softening, with the fruits possessing lower amounts of ABA and ethylene through the downregulation of NCED, ACS, and ACO, as well as of pectin-modifying enzymes [107].

### 5.5. Melatonin and Ethylene

Melatonin triggers ethylene production and controls a significant portion of the process by exerting control over a number of ripening factors, including RIN, CNR, NOR, and AP2a. The genes responsible for producing carotenoid biosynthesis are activated by melatonin treatment in tomatoes. Melatonin also increased the transcription of the ACO and ACS genes as well as transducing elements (EIL1, EIL3, and ERF2). A differential proteomic examination of tomato fruits revealed that melatonin has a considerable impact on several proteins implicated in the pathways connected to ripening [108]. Melatonin application on the seeds of tomatoes increased the production of fruits with higher concentrations of ASA, lycopene, galactose, citric acid, and calcium. During this time, the N, P, Mg, Cu, Zn, Fe, and Mn levels were reduced, and ripening and flavor improved [109]. The application of melatonin on strawberries exhibited progress in postharvest indicators. Melatonin application prolonged fruit shelf life and increased the number of bioactive substances (antioxidants, flavonoids, and phenolic acids), and improved fruit quality indicators [110]. It activates the genes for ethylene production enzymes (ACO and ACS) in plants [108].

### 5.6. Melatonin and Salicylic Acid (SA)

Biological stress responses in plants are regulated by SA [63,111]. In general, the production of SA, JA, and ethylene is increased with the application of melatonin. Previous studies [93] revealed the crucial role of melatonin in plants’ defense against biotic stress. Regarding SA, it was found that *Pseudomonas syringae* DC3000-infected Arabidopsis caused an increase in melatonin levels and SA. Melatonin and SA levels were decreased in SNAT knockout mutants, and they were also more vulnerable to pathogen attacks [112]. By increasing the synthesis of SA and NO, exogenously administered melatonin protected plants from the tobacco mosaic virus. Relative viral RNA and virus titer levels were decreased by melatonin therapy [75].

### 5.7. Melatonin and Jasmonic Acid (JA)

Plant hormones known as jasmonates include methyl jasmonate (MeJA) and jasmonic acid (JA). They control a wide range of features related to plant development, growth, and stress reactions. So, the genes, proteins, and metabolites which are involved in the defense system of the plants are upregulated by jasmonates [113]. Recent research suggests that the melatonin—JA interaction is quite complex. For instance, melatonin therapies affect JA levels in abiotic stress trials, though not in an obvious way. Melatonin promotes a decrease in JA biosynthesis and its level in *B. napus* growing under salt stress. Additionally, it triggers the production of JAZ proteins (repressor proteins in the JA signaling pathway), which suppress the response mediated by JA. As a result, there is a reduced response to JA, which enhances plant salt tolerance and development [96]. Nevertheless, a rise in JA levels in tomatoes growing under drought stress has been documented [87].

Plant hormones known as jasmonates include methyl jasmonate (MeJA) and jasmonic acid (JA). They control a wide range of features related to plant development, growth, and stress reactions. So, the genes, proteins, and metabolites which are involved in the defense system of the plants are upregulated by jasmonates [113]. Recent research suggests that the melatonin—JA interaction is quite complex. For instance, melatonin therapies affect JA levels in abiotic stress trials, though not in an obvious way. Melatonin promotes a decrease in JA biosynthesis and its level in *B. napus* growing under salt stress. Additionally, it triggers the production of JAZ proteins (repressor proteins in the JA signaling pathway), which suppress the response mediated by JA. As a result, there is a reduced response to JA, which enhances plant salt tolerance and development [96]. Nevertheless, a rise in JA levels in tomatoes growing under drought stress has been documented [87].

Although melatonin’s impact on JA levels can fluctuate, it seems that melatonin slows or reverses the JA response through JAZ protein production. Depending on whether the stressor is biotic or abiotic, it is probable that the melatonin–JA interaction varies. High melatonin doses (0.1 and 1.0 mM) decreased primary root development in a recent study using Arabidopsis. Additionally, the transcript accumulation of the genes responsible for the production of JA, brassinosteroids, and CK was decreased. In contrast, genes responsible for the production of ethylene, strigolactones, and GA were increased [114].

### 5.8. Melatonin and Brassinosteroids (BRs)

BRs are steroid hormones that affect cell lengthening and division to regulate several aspects of plant growth and development. The protective role of BRs in shielding plants from abiotic stress is well documented [115]. The production of BRs in rice is regulated by the application of melatonin via an increase in the transcript accumulation of BR producing genes. *Skotomorphogenesis*, a response to darkness, appears to be influenced by melatonin [116]. High melatonin levels reduced the expression of genes involved in IAA and BR production, which prevented the growth of Arabidopsis roots [114].

## 6. Melatonin Receptors and Signaling in Plants

Numerous studies have reported that melatonin is found in a variety of plant species and that it plays a pivotal role in a variety of physiological processes, such as growth promotion, rooting induction, seed germination, optimizing photosynthetic efficiency, leaf water/CO_2_ exchange, regulating the internal biological clock, and ripening/senescence processes. Moreover, it also functions as an endogenous biostimulator against biotic or abiotic stresses. Due to a lack of understanding of its receptor, the precise function and signaling pathway of phytomelatonin are primarily unknown.

Recently, Wei, et al. [117] identified the first phytomelatonin receptor (CAND2/PMTR1) in *A*. *thaliana*. They reported that the G subunit-mediated H_2_O_2_ generation and Ca_2_^+^ flow dynamics govern stomatal closure through the CAND2/PMTR1-dependent phytomelatonin signaling and suggest that phytomelatonin is a novel phytohormone that regulates stomatal closure via the H_2_O_2_ and Ca_2_^+^ signaling transduction cascade mediated by CAND2/PMTR1. On the other hand, melatonin-induced H_2_O_2_ production and stomatal closure were not supported by some of the previous reports and suggested that melatonin receptors may be required to activate the MAPK cascade [118]. More recently, Lee and Back [119] again examined CAND2, and suggested that the integrity of CAND2 as a melatonin receptor requires detailed studies because their confocal microscopy analysis revealed that the CAND2 protein is localized in the cytoplasm rather than the plasma membrane. They used two *A. thaliana* CAND2 knockout mutant lines, SALK 071,302 (CAND2-1) and SALK 068848, in genetic investigations to further examine the function of CAND2-2. They discovered that melatonin-mediated mitogen-activated protein kinase (MAPK) activation was not eliminated in the CAND2 mutant lines, nor did melatonin-mediated defense gene induction (such as GST1) change in comparison to that in the wild-type Col-0. Thus, the discovery of CAND2 as a plant melatonin receptor raises significant concerns about the general function of melatonin as a strong antioxidant [120].

## 7. Conclusions and Future Prospects

The pragmatic role of melatonin in the growth and development of plants, as well as its potential as a regulatory molecule in circadian rhythms and photoperiodicity, has received great attention in recent times. The circadian oscillator in plants is capable of adjusting the phases of a range of biological processes under varying conditions. These processes include gene expression, metabolic regulation, protein stability, and different other processes related with routine cycles (photoperiods/seasonal). The robust circadian regulation enhances plant growth and development and influences photosynthesis and crop productivity, even under biotic and abiotic stress conditions [121]. In the present review, we reported the main function of melatonin in the growth and development of plant organs such as rooting induction, seed germination, and optimizing photosynthetic efficiency. Similarly, we have focused on the role of melatonin under biotic and abiotic stress conditions. Additionally, we discussed the crosstalk of melatonin with other phytohormones under normal and stressful conditions and reported melatonin receptors and signaling in different plants.

Melatonin is reported for its role in the expression of different genes and regulation of related factors that halt or revert the unwanted effect of stressful conditions on the growth and development of the plants. Therefore, the role of melatonin is pivotal under oxidative damage and the expression of many stress-responsive genes. There are still many questions to be answered, but melatonin has been suggested as a common effector under stressful conditions, suggesting that melatonin may be an important regulator of growth and defense mechanisms. Similarly, in terms of future challenges, all aspects of melatonin must be considered for detailed research, including its gene expression, the enzymes involved in the biosynthesis of melatonin, and its metabolism. According to some recent researchers, melatonin is thought to be produced in the mitochondria and chloroplasts. Although evidence suggests that roots have the greatest melatonin level of the entire plant—probably because it is generated there—this has yet to be proved. Additionally, it is important to look at the presence of melatonin in the xylem and/or phloem parts of plants. Due to a lack of knowledge regarding the receptor, the receptor-mediated signaling cascade, and the actions of phytomelatonin, the question of whether it is a hormone is still debatable. However, some data suggest that phytomelatonin signaling regulates stomatal closure, which has led some researchers to hypothesize that phytomelatonin acts as a phytohormone via the CAND2/PMTR1-mediated signaling pathway to control stomatal closure. Conclusively, melatonin’s capacity to sustain plant growth and development under a variety of biotic and abiotic challenges has uncovered an exciting new field of research and a way forward for sustainable agriculture.

## Figures and Tables

**Table 1:** Role of exogenously applied melatonin in plant–microbe interactions.

Plant	Pathogen	Mechanism	Effect	References
*Arabidopsis thaliana*, *Nicotiana benthamiana*	*Pseudomonas syringae*	Expression of pathogenesis-related genes	Inhibition of pathogenic growth	[82]
*A. thaliana*	*P. syringae*	Melatonin-mediated innate immunity in SA- and (Nitric oxide) NO-dependent pathways	Disease resistance	[83]
*A. thaliana*	*P. syringae*	NO and melatonin levels in leaves and defense-related genes	Improvement of disease resistance	[79]
*Musa acuminata*	*Fusarium oxysporum*	Resistance induced via regulating the expression of MaHSP90s gene	Improvement of disease resistance	[84]
*Fragaria ananassa*	*Botrytis cinerea, Rhizopus stolonifer*	H_2_O_2_ levels and antioxidant enzyme activities	Reduction in postharvest decay in stored strawberry fruits	[85]
*Citrullus lanatus*	*Podosphera xanthii, Phytophthora capsici*	Upregulation of PTI- and ETI-associated genes	Disease resistance	[81]
